# Direct Charge Trapping Multilevel Memory with Graphdiyne/MoS_2_ Van der Waals Heterostructure

**DOI:** 10.1002/advs.202101417

**Published:** 2021-09-09

**Authors:** Jialing Wen, Wenhui Tang, Zhuo Kang, Qingliang Liao, Mengyu Hong, Junli Du, Xiankun Zhang, Huihui Yu, Haonan Si, Zheng Zhang, Yue Zhang

**Affiliations:** ^1^ Academy for Advanced Interdisciplinary Science and Technology Beijing Advanced Innovation Center for Materials Genome Engineering University of Science and Technology Beijing Beijing 100083 P. R. China; ^2^ Beijing Key Laboratory for Advanced Energy Materials and Technologies School of Materials Science and Engineering University of Science and Technology Beijing Beijing 100083 P. R. China

**Keywords:** bilayer memory, direct charge trapping, graphdiyne, multilevel memory, van der Waals coupling

## Abstract

Direct charge trapping memory, a new concept memory without any dielectric, has begun to attract attention. However, such memory is still at the incipient stage, of which the charge‐trapping capability depends on localized electronic states that originated from the limited surface functional groups. To further advance such memory, a material with rich hybrid states is highly desired. Here, a van der Waals heterostructure design is proposed utilizing the 2D graphdiyne (GDY) which possesses abundant hybrid states with different chemical groups. In order to form the desirable van der Waals coupling, the plasma etching method is used to rapidly achieve the ultrathin 2D GDY with smooth surface for the first time. With the plasma‐treated 2D GDY as charge‐trapping layer, a direct charge‐trapping memory based on GDY/MoS_2_ is constructed. This bilayer memory is featured with large memory window (90 V) and high degree of modulation (on/off ratio around 8 × 10^7^). Two operating mode can be achieved and data storage capability of 9 and 10 current levels can be obtained, respectively, in electronic and opto‐electronic mode. This GDY/MoS_2_ memory introduces a novel application of GDY as rich states charge‐trapping center and offers a new strategy of realizing high performance dielectric‐free electronics, such as optical memories and artificial synaptic.

## Graphical abstract

A graphdiyne (GDY)/MoS_2_ bilayer memory without any blocking layer is constructed. Through controllable oxygen plasma treatment, relatively large‐area and smooth 2D GDY with additionally introduced C—O and C═O is obtained, which enables excellent van der Waals coupling with MoS_2_ and allows for constructing the bilayer memory. With more introduced states, multilevel and dual operating mode is achieved.

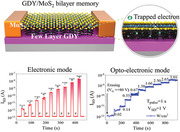

## 1 Introduction

With the coming age of Internet of things, high‐capacity data storage devices are pivotal components to meet the demand of huge surge of the information. To this end, dimensional scaling of the unit cell has been being the main technique which, however, remains a bottleneck for conventional silicon‐based memories. Owing to the ultra‐thin thickness, free of dangling bonds and highly compatibility with existing devices, 2D materials (2DMs) have been utilized as promising materials for device scaling down. The 2DMs‐based memory has novel memory functions, including multilevel storage^[^ ^1^, ^2^ ^]^ and multifunctional memory.^[^ ^3^, ^4^ ^]^ The reported 2DMs‐based memories are mainly of two structures which are floating‐gate structures^[^ ^5^, ^6^, ^7^, ^8^, ^9^ ^]^ and direct charge‐trapping structures.^[^ ^10^, ^11^, ^12^, ^13^ ^]^ Direct charge‐trapping memories are tailor‐made architecture that is designed from the unique nature of 2DMs, which is of only one or two layers without interlayer materials providing a promising way for next‐generation memories. Despite the novel structure and outstanding properties, the development of direct charge‐trapping memories based on 2DMs is still at incipient stage, far behind that of their floating‐gate memory counterparts. In such devices, localized electronic states, provided by chemical groups such as Si—O bonds,^[^ ^11^ ^]^ O dangling bonds,^[^ ^13^ ^]^ and defects in h‐BN,^[^ ^12^ ^]^ are served as charge‐trapping centers to trap electrons or holes, which is also famous as the interlayer states that are able to tailor the 2DMs.^[^ ^14^ ^]^

To further advance direct charge‐trapping memory with two or more operating mode, introducing a material with ample hybrid states is highly preferred. Graphdiyne (GDY), a new 2D carbon‐allotrope material consisting of hybrid states of sp^2^ from benzene rings and sp from acetenyl groups, has been synthesized in recent years^[^ ^15^, ^16^, ^17^ ^]^ and has shown great performances in various applications.^[^ ^18^, ^19^ ^]^ The charge trapping ability of graphdiyne has also been reported in many devices, like infrared detector,^[^ ^20^ ^]^ high‐performance UV detector,^[^ ^21^ ^]^ and resistive random access memory devices.^[^ ^22^ ^]^ However, 2D GDY with relatively large‐area and smooth surface is difficult to obtain due to its sophisticated growth process, which poses a restraint on its desirable van der Waals coupling with other 2D materials. Moreover, pure GDY is semiconducting which is not readily applicable to nondielectric memory.

Here, we propose a novel strategy to construct bilayer GDY/MoS_2_ direct charge‐trapping memory without any dielectric layer by using mild‐oxygen‐plasma‐treated GDY. Through controllable oxygen plasma treatment, relatively large‐area and smooth 2D GDY with additionally introduced C—O and C═O bonds was obtained, which forms an excellent van der Waals coupling with MoS_2_ and the treated GDY with many C—O and C═O bonds was changed to be insulating (Figure S1, Supporting Information) which allows for direct integration with MoS_2_ with no need of any dielectric layer. Moreover, the hybrid states of GDY with different chemical groups provide more localized states for trapping carriers, which enables more efficient control over the MoS_2_ of channel. The bilayer GDY/MoS_2_ memory is featured with dual operating mode and large memory window (90 V) and high degree of modulation (on/off ratio around 8 × 10^7^). The high modulation degree makes it possible for nine distinct storage states controlled by gate pulses and ten states by laser pulses. This multilevel memory is an effective and low‐cost way to increase storage capacity from 2*^n^* to 9*^n^* of electric signal or to 10*^n^* of photonic signal.

## 2 Results

### 2.1 Plasma Treatment of Graphdiyne and Device Construction

**Figure 1:**
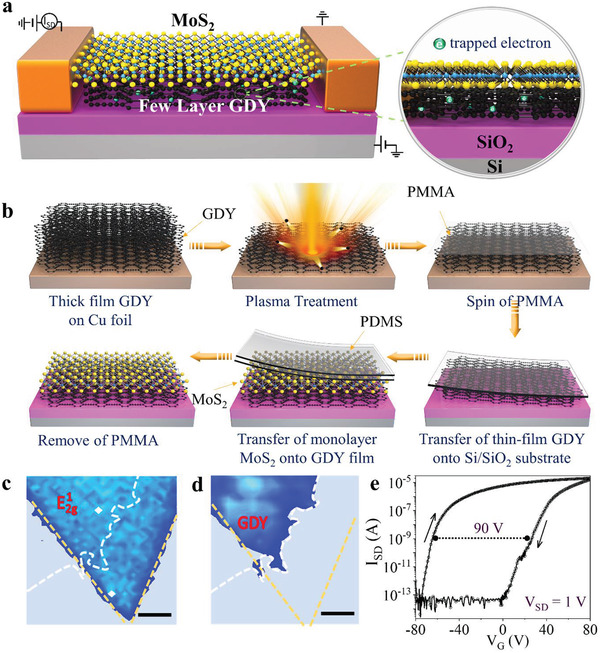
Memory transistor based on GDY/MoS_2_ heterostructure. a) Overview diagram of GDY/MoS_2_ memory device. b) Schematic illustration of the procedure of constructing GDY/MoS_2_ memory device. c) Raman mapping images of monolayer MoS_2_ on top of GDY at range of $E_{2g}^{1}$. d) Raman mapping of device at range of GDY. The scale bars in (c) and (d) are 5 µm. e) Transfer characteristic curve of memory device which includes positive sweep from +80 to −80 V and negative sweep from −80 to +80 V.

**Figure** **1**a shows a schematic illustration of the GDY/MoS_2_ bilayer direct charge‐trapping memory. In this device, the Si wafer supporting with a 300 nm thick SiO_2_ layer acted as the back gate. Figure 1b demonstrates the schematic procedure of GDY processing and MoS_2_/GDY multilevel memory constructing. Original GDY was synthesized via a cross‐coupling reaction on the surface of copper^[^ ^23^ ^]^ and was of ≈1 µm thick with undulate surface (Figure S2a,b, Supporting Information). The chemical liquid grown GDY is uncontrollable on its thickness and roughness because of free rotation of alkyne—aryl single bonds^[^ ^24^ ^]^ which creates a barricade on its formation of a desirable van der Waals coupling with other 2D materials. Therefore, we utilized mild oxygen plasma treatment method to decrease its thickness and smooth the surface (see details in the Experimental Section). Plasma treatment is an effective way to control layer thickness^[^ ^25^ ^]^ or to tune properties of graphene‐like materials.^[^ ^26^, ^27^, ^28^, ^29^ ^]^ The obtained GDY has a flat area of 100 µm in length as identified by scanning electron microscopy (SEM; Figure S2c, Supporting Information) proving that the plasma treatment is a feasible method to produce large area GDY with smooth surface (Figure S3, Supporting Information). According to atomic force microscopy (AFM) in Figure S2d in the Supporting Information, GDY was of ≈4 nm thick, revealing the 10‐layers nature of the film.^[^ ^30^ ^]^ Raman spectrum can provide useful methods to confirm structure of GDY. Four typical Raman peaks, including G, D, and two conjugated diine links, could be observed with slightly decreased intensity, suggesting the plasma treatment is unharmful to the GDY structure which is left (Figure S6, Supporting Information).^[^ ^23^, ^31^ ^]^

After treated by mild plasma, GDY was transferred onto Si/SiO_2_ substrate. Monolayer MoS_2_ was prepared by chemical vapor deposition (CVD, see details in Experimental Section and Figure S4, Supporting Information) and was then stacked onto GDY using polymethyl methacrylate (PMMA)‐assisted transfer method. The Cr/Au (10 nm/50 nm) electrodes were deposited after patterned via electron beam lithography. Figure 1c,d shows the Raman mapping spectra of the MoS_2_/GDY bilayer heterostructure over the area depicted by SEM (Figure S6a, Supporting Information). Figure 1c shows the peak frequency mapping for the $E_{2g}^{1}$which guarantees MoS_2_ layers on the GDY are of a uniform distribution and can exclude the impact of strain caused by surface roughness.^[^ ^32^, ^33^, ^34^ ^]^ After mild plasma treatment, we observed weaker peaks of GDY. However, in Raman mapping of heterostructure, intensity of GDY beneath MoS_2_ was observed to be strengthened by up to 50 times (at G peaks) than that of GDY without MoS_2_ as shown in Figure 1d and Figure S6d in the Supporting Information. This phenomenon is due to the MoS_2_‐enhanced Raman scattering effect.^[^ ^35^, ^36^, ^37^ ^]^ Through enhanced Raman signals and uniform distribution of intensity, the structure and uniformity of GDY film were further confirmed.

Figure 1e shows the transfer characteristic curves of the device with dual sweep gate voltage (from +80 to −80 V and then reversed). A large hysteresis window up to 90 V is observed due to the charge injection and trapping in GDY, which demonstrates memory function can be developed in this device and thusly the excellent van der Waals coupling between GDY and MoS_2_. Furthermore, multilevel memory was also available as the on/off current ratio at *V*_g_ = 0 was ≈8 × 10^7^. To exclude other experimental elements that might influence the result, transfer curve of monolayer MoS_2_ FET on SiO_2_ was also performed in the same condition. The corresponding hysteresis memory window is only 20 V and exhibits depletion characteristics (Figure S6, Supporting Information) indicating limited charge trapping originated from absorbed oxygen and water molecules on the interface of MoS_2_/SiO_2_.^[^ ^38^ ^]^ The hysteresis window and on/off ratio statistics were performed on 19 GDY/MoS_2_ bilayer devices (Figure S7, Supporting Information) indicating highly uniform device quality. Therefore, this manifests the accuracy of our results and the memory performance of MoS_2_/GDY bilayer heterostructure originated from trapping of GDY layer.

### 2.2 Mechanism of Memory Device

**Figure 2:**
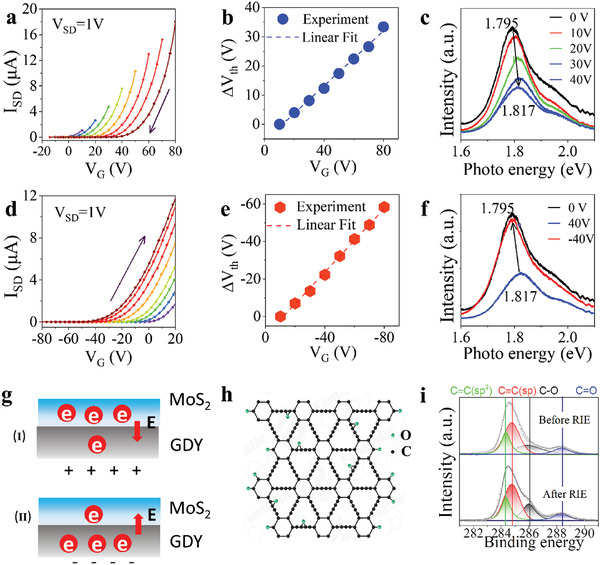
The charge trapping mechanism of the memory transistor. a) Transfer curves measured with different positive start gate at a swept rate of 0.5 V s^−1^. b) Variation of the hysteresis voltage Δ*V*_th_ dependent on the positive gate voltage. Here, Δ*V*_th_ is the difference of *V*_ON_ between *V*_g_ and *V*_g_ = 10 V. c) PL spectrum evolution after reset operation using various positive gate. d) Transfer curves with different negative gate voltage at a rate of 0.5 V s^−1^. e) Variation of the hysteresis voltage Δ*V*_th_ dependent on the negative gate voltage. Here, Δ*V*_th_ is the difference of *V*_ON_ between *V*_g_ and *V*_g_ = −10 V. f) PL spectrum after reset and programing operation. g) Schematic diagram of the memory device at two key states, I: reset, positive gate voltage realizes tunneling into GDY; II: programing, negative control gate voltage release charges. h) Schematic illustration of GDY structure after mild oxygen plasma treatment. The green and black circle is O and C element, respectively. i) XPS spectra of GDY, the four peaks are attributed to C═C, C≡C, C—O, and C═O.

The mechanism of gate‐controlled memory function of MoS_2_/GDY nonvolatile memory heterostructure device was investigated by hysteresis behavior. **Figure** **2**a depicts the transfer characteristics of the device measured with different positive *V*_g_ from +10 to +80 V. When back gate sweeps from a positive voltage, electrons in intrinsically n‐type MoS_2_ are accumulated and then trapped in GDY. The localized electrons can weaken the external electric field exerting on MoS_2_. As *V*_g_ starts more positively, more electrons are collected by GDY, and the turn‐on voltage (*V*_ON_) is positively shifted which leads to the drain current at *V*_g_ = 0 V decreased gradually to 10^–13^ A. It is noted that the electrons are stored in GDY even after removing the gate voltage which produces a negative electric field to MoS_2_ and suppresses free carriers in the channel. Therefore, after a positive gate voltage pulse, the accumulated electrons are trapped in GDY and an ultra‐low drain current at *V*_g_ = 0 V can be obtained. This operation is defined as “reset,” as illustrated schematically in Figure 2g‐I. By contrast, when transfer characteristics are measured with various negative *V*_g_ from −10 to −80 V after a +80 V reset process, we observe that the turn‐on voltage (*V*_ON_) was negatively shifted (Figure 2d). These indicated that more electrons in GDY are transferred back to MoS_2_ because of the increasing negative *V*_g_. The increased drain current at *V*_g_ = 0 V also confirms the effective transfer of electrons. This procedure is termed as “programming,” as shown in schematic diagram in Figure 2g‐II. The observed asymmetry shifts of *V*_ON_ shown in Figure 2b–e, implying various energy barrier of trapping and detrapping electrons for GDY, which might be attributed to different mechanism between trapping and detrapping of electrons, and we will discuss this point later. To further reveal gate‐controlled memory of the devices, output behavior is investigated at various gate pulse voltage and integration time. It is validated that the readout current depends linearly on gate pulse voltage which means that the current can be tuned by selected gate pulse voltage according to the specific requirements (Figure S8, Supporting Information). We also confirm that the readout current is proportional to integration time (Figure S9, Supporting Information).

To demonstrate the electron transfer between the GDY and MoS_2_, photoluminescence (PL) spectrum was measured after reset (Figure 2c) and programming (Figure 2f), which further confirmed the great van der Waals coupling between GDY and MoS_2_. Figure 2c shows PL spectrum of the memory device after reset process. An obvious blue shift of the PL peak from 1.795 to 1.817 eV can be observed indicating the decrease of electron density in monolayer MoS_2_. The quenching intensity of PL spectrum was also observed which may be due to the interfacial transfer of photogenerated holes to trapping layer and then couple with trapped electrons in GDY,^[^ ^39^, ^40^, ^41^ ^]^ as depicted in Figure S10a in the Supporting Information. Therefore, after the positive gate pulse, the PL intensity did not increase like that of continuously positive‐gated MoS_2_^[^ ^42^ ^]^ or p‐doped MoS_2_.^[^ ^43^, ^44^ ^]^ Once a negative voltage pulse is exerted, the density of electrons in MoS_2_ is recovered which can be manifested by redshift of PL peaks and the stronger intensity, as illustrated in Figure S10b in the Supporting Information.

The as‐confirmed efficient charge transfer between GDY and MoS_2_ can only be achieved until an efficient van der Waals coupling between them is developed, which cannot be realized otherwise. We attribute the charge trapping performance to the hybrid states of GDY including C—O and C═O bonds introduced by mild oxygen plasma treatment and the excellent van der Waals coupling between GDY and MoS_2_ (Figure 2h). The unique property of GDY is numerous molecular‐scale active alkyne units that may be the ideal platform for charge‐trapping, since they can act as charge‐attracting centers. More importantly, these active alkyne units can be easily opened up by external stimuli such as oxygen plasma. X‐ray photoelectron spectroscopy (XPS) spectrum was measured before and after mild oxygen plasma treatment. As shown in XPS spectra (Figure 2i), the deconvolution of C 1s peak resolved four peaks at 284.3, 284.8, 286.0, and 288.4 eV, which could be assigned to C═C, C≡C, C—O, and C═O, respectively.^[^ ^24^, ^45^ ^]^ Compared to the XPS spectrum of the original GDY, the binding energy of C═C, C≡C, and C═O after treatment has no obvious difference which suggests the mild oxygen plasma treatment dose not destroy the hybrid states of GDY. A little increase of binding energy at 286.0 eV is observed indicating more oxygen‐containing functional groups are introduced. It is noted that oxygen‐containing functional groups are easily introduced, C—O and C═O, which exhibit strong polar interaction, hence causing a direct modulation of the energy band structure in monolayer MoS_2_. Explicitly, this results in uniformly distributed local potential fluctuations that are able to trap electrons energetically.^[^ ^4^, ^6^, ^46^, ^47^, ^48^, ^49^ ^]^ Raman spectrum is also performed (Figure S6, Supporting Information) to profile the bonding structure of GDY film. Two obvious peaks at 1929 and 2202 cm^–1^ are observed which further validates the alkyne bonds of mild‐treated GDY. We also found that the band intensity ratio of *I*_D_/*I*_G_, which reflects graphitization degree and defects, is decreased from 0.63 to 0.39 indicating the mild oxygen plasma treatment can also remove impurities and amorphous structures of liquid‐grown GDY. Therefore, the numerous C—O and C═O bonds of GDY, as Figure 2h shows, were expected to enable charge trapping ability of the devices mutually. As oxidization always bring about conductivity reduction and introducing charge trapping center,^[^ ^50^ ^]^ our oxygen‐plasma‐treated GDY is nonconducting which makes the GDY possible to maintain electrons and therefore the memory without dielectric layers. The reason is that the highest valance band and the lowest conduction band are both doubly degenerated and consist mainly of the overlap 2p*_z_* carbon atomic orbitals, which exactly form the numerous active alkyne units of GDY and provide charge carriers since GDY possesses both sp‐ and sp^2^‐hybridized carbon atoms. The oxygen plasma treatment we employed to GDY is readily able to break up the active alkyne units which hamper the original source of the charge carriers. Therefore, the oxygen plasma treatment is able to compromise the conductivity of the GDY. The numerous alkyne bonds of GDY are very active as positive‐charge‐attracting magnet, which are legitimately considered as negative‐charge‐repelling centers as well. These alkyne bonds would repel the electrons to some extent, forming an additional barrier for trapping electrons. Thus, it is relatively more difficult for electrons trapping into GDY than detrapping out of GDY. As a result, under the same magnitude of gate pulse, the additional barrier from alkyne leads to relatively small amounts of trapped electrons and thusly lower doping degree in the trapping process, and eventually the asymmetric shift of *V*_ON_ as shown in Figure 2b–e.

### 2.3 Electrically Controlled Multilevel Memory Performance

**Figure 3:**
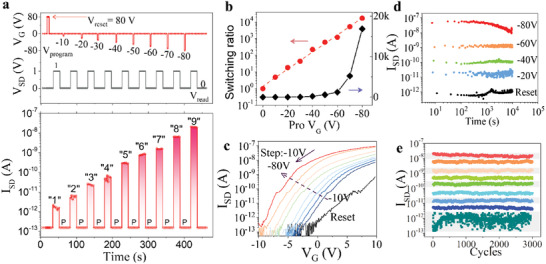
Electrically controlled memory behavior of the GDY/MoS_2_ memory device. a) Schematic illustration of the memory operation procedure at different programming gate from −10 to −80 V with step of −10 V. The correspond logarithmic readout current of the procedure is shown and clear 9 level is generated. b) Switching ratio corresponding with *V*_pro_ in linearity scale and logarithmic scale. c) Transfer characteristics of the memory device after reset operation and then the programming (*V*_pro_ from −10 to −80 V, −10 V step) operations. d) Retention time for device to kept in dark condition without electric field, the ratio remains the same after 10^4^ s. e) Cyclic property of the memory device, including 3000 cycles of 9 readout levels. The MoS_2_ transistor has a *L* = 3 µm, *W* = 10 µm.

In order to verify the performance of multilevel programmable electronic memory, we simulate a dynamic multilevel memory operation by modulating programming gate pulse (*V*_pro_) after a positive resetting gate pulse (*V*_reset_) of 80 V, as shown in **Figure** **3**a. Reset operation is realized with an 8 s positive gate pulse and the programming operation is conducted with 1 s negative gate pulse. The source was grounded and the drain was biased by 1 V. After reset, a stable off‐current around 10^–12^ A was obtained at *V*_g_ = 0 representing the memory was initialized. When negative programming gate pulse (*V*_pro_) increases from −10 to −80 V, the read‐out current increases step by step. Switching ratios, dividing each programming current by lowest off‐current, were extracted and displayed in Figure 3b. It is shown that the switching ratio in exponential form increases from 1 to 10^4^ and shows a linear relationship with *V*_pro_. Figure 3c shows the transfer characteristics evolution of MoS_2_/GDY memory device under different programming gate pulse (*V*_pro_, from −10 to −80 V) after a reset. The *V*_ON_ shifted gradually to the negative side during process which indicated a significant gate‐tunable reversion of MoS_2_ from p‐doped to slightly n‐doped.

Retention time and cyclic reset/release endurance of MoS_2_/GDY memory device were investigated as to evaluate its practical application. Retention performance of five states was investigated and highly reliable retention performance was achieved as depicted in Figure 3d. It is shown that the life time of trapped charges in GDY can be maintained and negligible degradation of readout current was observed over 10^4^ s, and the high retention capability depends on efficient van der Waals coupling between MoS_2_ and smoother GDY treated via 500 s oxygen plasma (Figure S11, Supporting Information). The read of the current was done without back gate and under dark circumstances. In the endurance test, nine states of the device maintain high endurance performance for round 3000 cycles indicating a stable quality (Figure 3e). To define the vibration of nine states data, the results showed that these nine states had distinguishable ranges (Table S1, Supporting Information).

### 2.4 Opto‐Electrically Controlled Multilevel Memory Performance

**Figure 4:**
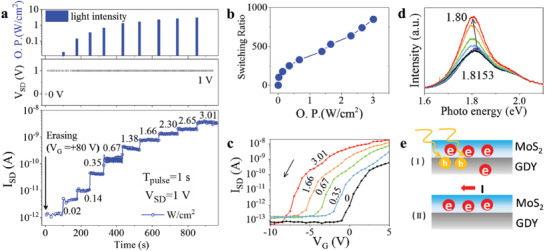
Opto‐electrically controlled GDY/MoS_2_ memory. a) Schematic illustration of the memory operation procedure at different light intensity after a reset voltage and the corresponding logarithmic readout current of the procedure. Clear 10 levels are generated. b) Switching ratio corresponding with Light_pro_ in linearity scale. c) Transfer characteristics of the memory device after reset operation and then the light programming operations. d) PL of the device after reset under different light programming power. e) Schematic diagram of the memory device during reset and light programming. The MoS_2_ transistor has a *L* = 3 µm, *W* = 10 µm.

Because of direct bandgap and strong light–matter interactions, monolayer MoS_2_ is picked to construct optoelectronic memory devices. **Figure** **4**a illustrates opto‐electrically controlled multilevel storage performance of MoS_2_/GDY bilayer memory. A positive voltage gate pulse is first processed to trap electrons in GDY which produced a low OFF current in memory as explained before. The electrical readout current of the memory device was then measured persistently, and 532 nm laser light pulses with different intensity from 0 to 3.01 W cm^−2^ for 1 s were conducted as shown in upper panel of Figure 4a. When the bias was applied continuously without illumination, the current persisted at one stable state and would not change until next light irradiation pulse, and all the readout currents for each Light_pro_ (*L*_pro_) showed good stable retention performance of up to 4000 s (Figure S12a, Supporting Information). For comparison, we performed the one time *L*_pro_ on pure MoS_2_ FET without charge trapping layer GDY (Figure S13, Supporting Information), the channel current is unable to be retained due to absence of charge trapping layer. Consequently, as irradiation intensity grew, the current of the memory device presented a stairs‐type to rise continuously, as shown in lower panel of Figure 4a. Also, the cyclic endurance for the opto‐electronic mode is characterized and it shows that the cyclic number is up to 3000 for each *L*_pro_ (Figure S12b, Supporting Information). To examine the intensity‐dependent states of memory, switching ratio was also extracted, revealing that the readout current was positively correlated with light intensity (Figure 4b).

To further illustrate the mechanism of opto‐electrically controlled multilevel function, electrical transfer curve and PL spectrum were measured. In Figure 4c, photoinduced transfer characteristics of the bilayer device were measured with 1 s laser pulse of 0, 0.35, 0.67, 1.66, 3.01 W cm^−2^ after reset gate pulse of +80 V. In this logarithmic plot, the transfer curves red‐shifted with increasing light intensity and the readout current at *V*_g_ = 0 V was increased. The photoinduced electron–hole pairs can be separated by the internal electric field induced by trapped electrons in GDY, leading to the combination of electrons in GDY and holes in MoS_2_, which can be considered as that the GDY releases the trapped electrons and thusly reduces the doping effect to the MoS_2_ channel as shown in Figure 4e.^[^ ^13^, ^51^, ^52^ ^]^ Eventually, the free electrons in MoS_2_ increased which is attested by the increasing readout current at *V*_g_ = 0 V. By releasing the trapped electrons in GDY, the readout current of MoS_2_ can be well controlled and have ten levels, which in fact realizes memorizing light information. It is noted that the difference between currents of last few light pulses is smaller as shown in Figure 4a, which means lesser amounts of electrons left in GDY and the resulting lower doping effect. Moreover, this process will not cease until the electric field generated from the remaining electrons is insufficient to initiate the driving process. At last, the current in MoS_2_ will saturate at a stable value below the original current value of MoS_2_ due to the remaining stored electrons as manifested in Figure 4a. Therefore, the stored electrons in GDY cannot be released completely solely via *L*_pro_, which results in a lower current value than in the case of *V*_pro_ (Figure 3a) and hence the smaller switching ratio. Moreover, to confirm what specific role of GDY in such opto‐electronic mode, PL measurements were carried out on the GDY/MoS_2_ heterostructure, the results show that the PL spectrum from GDY/MoS_2_ overlap region and MoS_2_ are similar with each other, indicating PL signal from the overlap region solely originated from MoS_2_. And the treated GDY has no PL contribution which has only noise background (Figure S14, Supporting Information).

When testing PL, green light of 532 nm wavelength can be taken as a light pulse. The black line in Figure 4d is obtained after a reset process. In the reset process, the photoexcited electron–holes pairs are mostly separated which largely decreases the recombination of electron–holes as depicted in Figure 4e. With continuous testing of PL, less separation of photoexcited electron–holes pairs happens for weaker electric field toward GDY which increases the recombination of photogenerated electron–holes pairs leading to the stronger signal of the PL curve. Red shift of the curve is also observed attesting increased electrons intensity.

## 3 Conclusions

In summary, we have demonstrated a dual mode multilevel memory of MoS_2_/GDY hetero‐bilayer for the first time, which could work in both electronic and opto‐electronic mode. Excellent van der Waals coupling is developed between 2D GDY and MoS_2_ using thickness controlled and surface smoothed GDY via mild oxygen plasma, which allows for the integration of 2D GDY with CVD grown monolayer MoS_2_ into a nondielectric van der Waals memory. The fabricated devices possess exceptionally high performance comparable to that of 2D charge trapping memory with FG structure and direct‐charge trapping memory. The fabricated van der Waals memory devices show excellent environmental data stability, long retention time (10^4^ s), and multilevel memory states with electronic (nine states) and opto‐electronic (ten states) operation modes. Our work is of great significance to extend the applications of GDY to multi‐level nonvolatile memories.

## 4 Experimental Section

### Preparation of Large‐Scale Graphdiyne

Few layers and flat graphdiyne nanosheet were achieved by mild oxygen irradiation. During the process of irradiation, oxygen of around 50 sccm was pumped into cavity to etch the thick layer of graphdiyne for around 500 s. Then the graphdiyne on copper flake was coated with PMMA and put onto 1 m mL^−1^ FeCl_3_ for 8 h to resolve copper, after which it was transferred onto SiO_2_/Si substrate and heated on 120 °C for 10 min to make sure the close contact of graphdiyne and SiO_2_ substrate, and then washed in acetone to remove PMMA. This simple method, reactive ion etching (RIE), removed upside thick layer of graphdiyne and a thin layer was revealed, undoubtedly providing a new way of creating thin‐layer graphdiyne.

### CVD Synthesis of Monolayer MoS_2_

With MoO_3_ (Sigma‐Aldrich, ≥99.5% purity) and sulfur (Sigma‐Aldrich, ≥99.5% purity) applied as precursor and reactant materials, respectively, MoS_2_ monolayers were grown onto SiO_2_/Si substrates by the oxygen‐assisted CVD method in furnace at 850 °C for 30 min. MoO_3_ powder was first placed in a quartz boat at the middle of quartz tube furnace with a 2 × 2 cm^2^ SiO_2_/Si substrates putting downface at top of the MoO_3_ powder. S powder was placed at the upstream of the tube and heated to 180 °C by heating belt. Ultrahigh purity argon flow of 500 sccm was used to carry S powder to react with MoO_3_. The experiments were implemented at a reaction temperature of 850 °C for 30 min with oxygen flow of 2 sccm. After cooling the sample, single‐layer MoS_2_ on SiO_2_ substrate was available.

### Fabrication of GDY/MoS_2_ Memory Device

The GDY was first transferred onto SiO_2_/Si substrate and then the CVD‐grown MoS_2_ was stacking onto the GDY partly with manipulated platform. The electrode contacts were drawn by a standard photolithography and Cr/Au (20/50 nm) electrodes, as source and drain electrodes, were deposited by thermal evaporation.

### Measurements

The morphology images of the film and device was analyzed by SEM in a FEI Quanta 3D. The thickness measurement of GDY and MoS_2_ was performed by AFM (Bruker Multimode Nanoscope IIID). Confocal Raman microscopic systems (Horiba Jobin Yvon HR800) with 532 nm laser were used to evaluate composition of thin‐layer materials. Measurements of the electrical properties were conducted using a Keithley 4200‐SCS Parameter Analyzer combined with a probe station placed at room temperature.

### Statistical Analysis

All measurements were carried in triplicate (*n* = 3) repeats and data results were presented via selecting the typical one. The employed statistical methods used to evaluate each experiment are detailed in the subsections of the Supporting Information describing every experimental technique, data processing, and analysis employed during the specific test.

## Conflict of Interest

The authors declare no conflict of interest.

## Author Contributions

J.L.W., W.H.T., and Z.K. contributed equally to this work. J.L.W., W.H.T., Z.K., Z.Z., and Z.Y conceived and designed the experiments. J.L.W. and B.S.L. performed the synthesis of MoS_2_ films by CVD. J.L.W. and H.H.Y. performed the plasma experiments and AFM measurements. J.L.W and M.Y.H carried out the materials characterization, device fabrication, and data collection. W.H.T., Z.K., J.L.D., and X.K.Z assisted in experiment analysis. J.L.W. and B.S.L. performed measurements of the Raman and PL. Z.Z. and Y.Z. initiated and supervised the project. All authors contributed to discussion and manuscript preparation.

## Supporting information

Supporting Information

## Data Availability

The data that support the findings of this study are available from the corresponding author upon reasonable request.
