# Supplementary material for: Direct Charge Trapping Multilevel Memory with Graphdiyne/MoS2 Van der Waals Heterostructure
Source: Adv Sci (Weinh). 2021 Sep 9;8(21):2101417. doi: 10.1002/advs.202101417 (PMC8564425; doi:10.1002/advs.202101417)
Supplement: Supplementary file 1 — Supporting Information [file ADVS-8-2101417-s001.pdf]

© 2021 Wiley-VCH GmbH

ADVANCED SCIENCE
Open Access

Supporting Information

for *Adv. Sci.,* DOI: 10.1002/advs.202101417

Direct Charge Trapping Multilevel Memory with Graphdiyne/MoS_2 Van der Waals Heterostructure

J. L. Wen, W. H. Tang, Prof. Z. Kang, Prof. Q. L. Liao, M. Y. Hong, Dr J. L. Du, Dr X. K. Zhang, H. H. Yu, Dr H. N. Si, Prof. Z. Zhang, Prof. Y. Zhang

Supporting Information for:

Direct Charge Trapping Multilevel Memory with Graphdiyne/MoS_2 Van der Waals Heterostructure

J. L. Wen, W. H. Tang, Prof. Z. Kang, Prof. Q. L. Liao, M. Y. Hong, Dr J. L. Du, Dr X. K. Zhang, H. H. Yu, Dr H. N. Si, Prof. Z. Zhang, Prof. Y. Zhang
Academy for Advanced Interdisciplinary Science and Technology, Beijing Advanced Innovation Center for Materials Genome Engineering, University of Science and Technology Beijing
Beijing 100083, People's Republic of China
E mail: zhangzheng@ustb.edu.cn; yuezhang@ustb.edu.cn;

J. L. Wen, W. H. Tang, Prof. Z. Kang, Prof. Q. L. Liao, M. Y. Hong, Dr J. L. Du, Dr X. K. Zhang, H. H. Yu, Dr H. N. Si, Prof. Z. Zhang, Prof. Y. Zhang
Beijing Key Laboratory for Advanced Energy Materials and Technologies, School of Materials Science and Engineering, University of Science and Technology Beijing
Beijing 100083, People's Republic of China

Jialing Wen, Wenhui Tang and Zhuo Kang contributed equally to this work.

Supporting Methods

Preparation of large area graphdiyne

Few layers and flat graphdiyne nanosheet is achieved by mild oxygen irradiation. During process of irradiation, oxygen of around 50 sccm is pumped into cavity to etch the thick layer of graphdiyne for around 500 s. Then the graphdiyne on copper flake was coated with PMMA and put onto 1 M/ml $FeCl_3$ for 8 h to resolve copper, after which it was transferred onto SiO_2/Si substrate and heated on 120 ℃ for 10 min to make sure the closely contact of graphdiyne and SiO_2 substrate, and then washed in acetone to remove PMMA. This simple method, RIE, removes upside thick layer of graphdiyne and a thin layer is revealed, undoubtedly providing a new way of creating thin-layer graphdiyne.

CVD synthesis of monolayer MoS_2

With MoO_3 (Sigma-Aldrich, ≥99.5% purity) and sulfur (Sigma-Aldrich, ≥99.5% purity) applied as precursor and reactant materials respectively, MoS_2 monolayers were grown onto SiO_2/Si substrates by the oxygen-assisted chemical vapor deposition (CVD) method in furnace at 850 °C for 30 min. MoO_3 powder was firstly placed in a quartz boat at the middle of quartz tube furnace with a 2 × 2 cm^2 SiO_2/Si substrates putting down face at top of the MoO_3 powder. S powder was placed at the upstream of the tube and heated to 180 ℃ by heating belt. Ultrahigh purity argon flow of 500 sccm were used to carried S powder to react with MoO_3. The experiments were implemented at a reaction temperature of 850 ℃ for 30 min with oxygen flow of 2 sccm. After cooling the sample, single-layer MoS_2 on SiO_2 substrate were available.

Fabrication of GDY/MoS_2 memory device

The graphdiyne was first transferred onto SiO_2/Si substrate and then the CVD-grown MoS_2 was stacking onto the graphdiyne partly with manipulate platform. The electrode contacts were drawn by a standard photolithography and Cr/Au (20 nm/50 nm) electrodes, as source and drain electrodes, were deposited by thermal evaporation.

Measurement

The morphology images of the film and device was analyzed by scanning electron microscopy (SEM in a FEI Quanta 3D). The thickness measurement of GDY and MoS_2 was performed by atomic force microscopy (AFM, Bruker Multimode Nanoscope IIID). Confocal Raman microscopic systems (Horiba Jobin Yvon HR800) with 532 nm laser were used to evaluate composition of thin-layer materials. Measurements of the electrical properties were conducted using a Keithley 4200-SCS Parameter Analyzer combined with a probe station placed at room temperature.

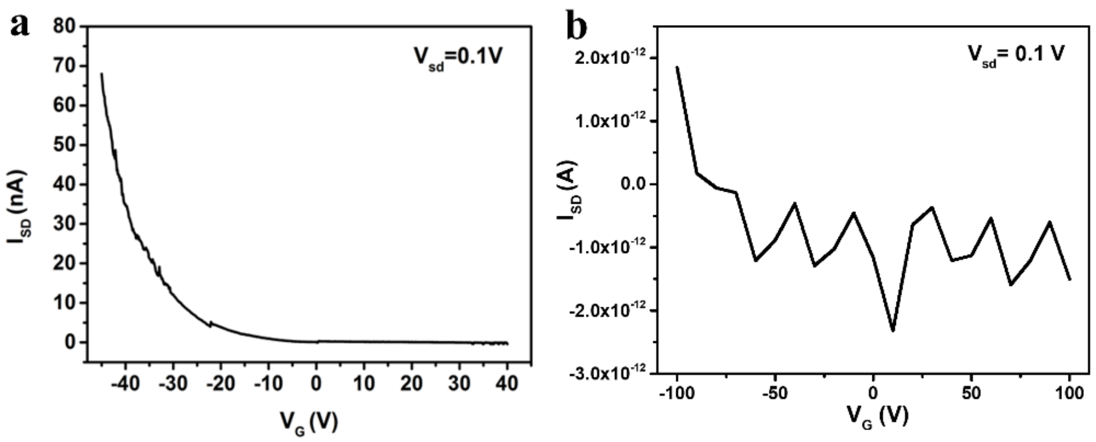

Supporting Figure 1. Transfer curves of GDY (a) before and (b) after oxygen plasma treatment, which show that the original GDY is a p-type semiconductor and the oxygen plasma treated GDY is non-conducting. GDY FET with L= 3 μm, W=10 μm.

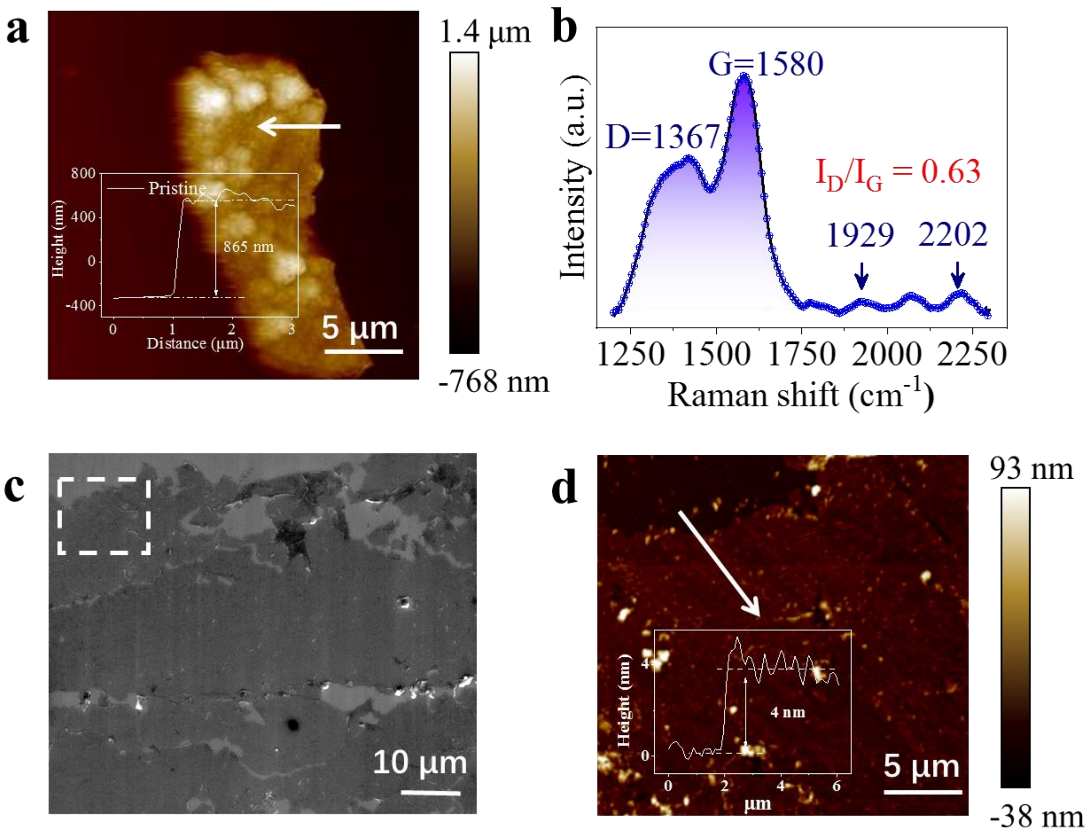

Supporting Figure 2. Comparision of original GDY and plasma-treated GDY. a) AFM topography of GDY flakes grown by cross-coupling reaction. The insert height profile was extracted from white line which showed that the thickness of original GDY is up to 865 nm. b) Raman spectrum of original GDY flakes. c) SEM images of GDY film transferred onto SiO_2 after treated by mild oxygen plasma treatment. GDY nanofilm of 100 μm was obtained. d) AFM topography of the dashed region area of **c** and the insert height profile was extracted from white line. The thickness of GDY is eliminated to 4 nm. It can be confirmed form the height profile that the oxygen plasma-treated GDY has thin and flat surface which is able to act as a base layer below MoS_2.

As dipicted in Supporting Figure 2b, Raman spetrum has shown the peaks of GDY. The G band at 1580 $cm^{-1}$and D band at 1367 cm^{-1} corresponds to the in-phase streching vibration and scissoring viration of sp^2 carbon in benzene The intensity ratio of D and G bands (D/G) indicates order and defects of GDY. Here, it is calculated to be 0.63. The peaks at 1929 and 2202 cm^{-1} is related to the vibration of conjuated diine

links (-C≡C-C≡C-).[1]The peaks at 2073 cm^{-1} is said to be the edges carbon or amorphous carbon.[2]

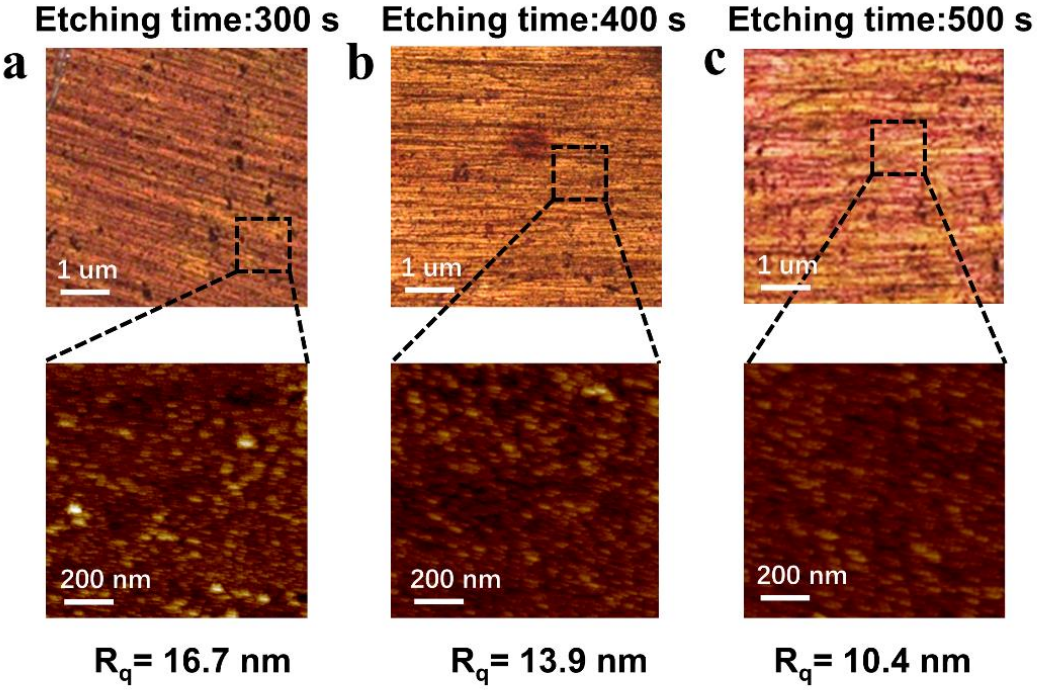

Supporting Figure 3. OM and AFM topography images of GDY after oxygen plasma treatment for various etching time. (a) 300 s, (b) 400 s and (c) 500 s etching time.

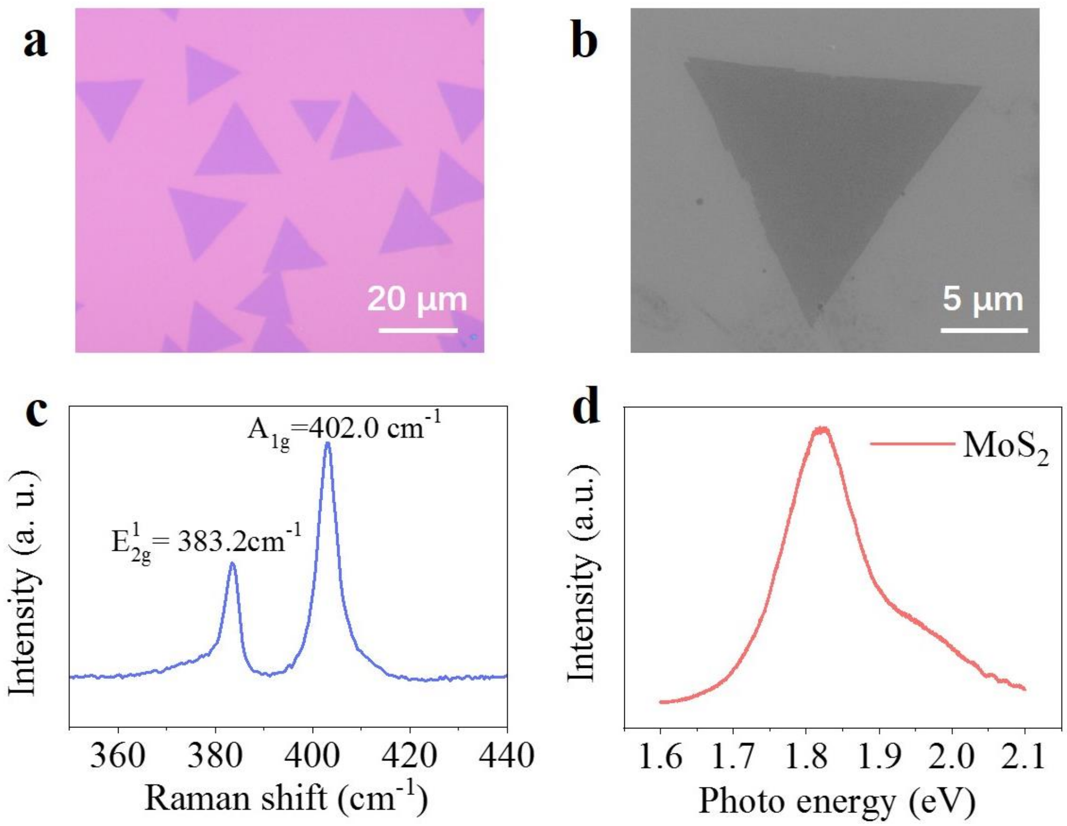

Supporting Figure 4. Characterization of CVD-grown MoS_2. a) Optical microscopy image of CVD-grown MoS_2. b) SEM image of MoS_2 transferred onto SiO_2. c) Raman spectrum of the CVD-grown MoS_2. The two phonon modes E^1_{2g} and A_{1g} at a separation of 19.9 cm^{-1} indicates the sample is of monolayer.[3] d) PL spectrum of the monolayer MoS_2.[4]

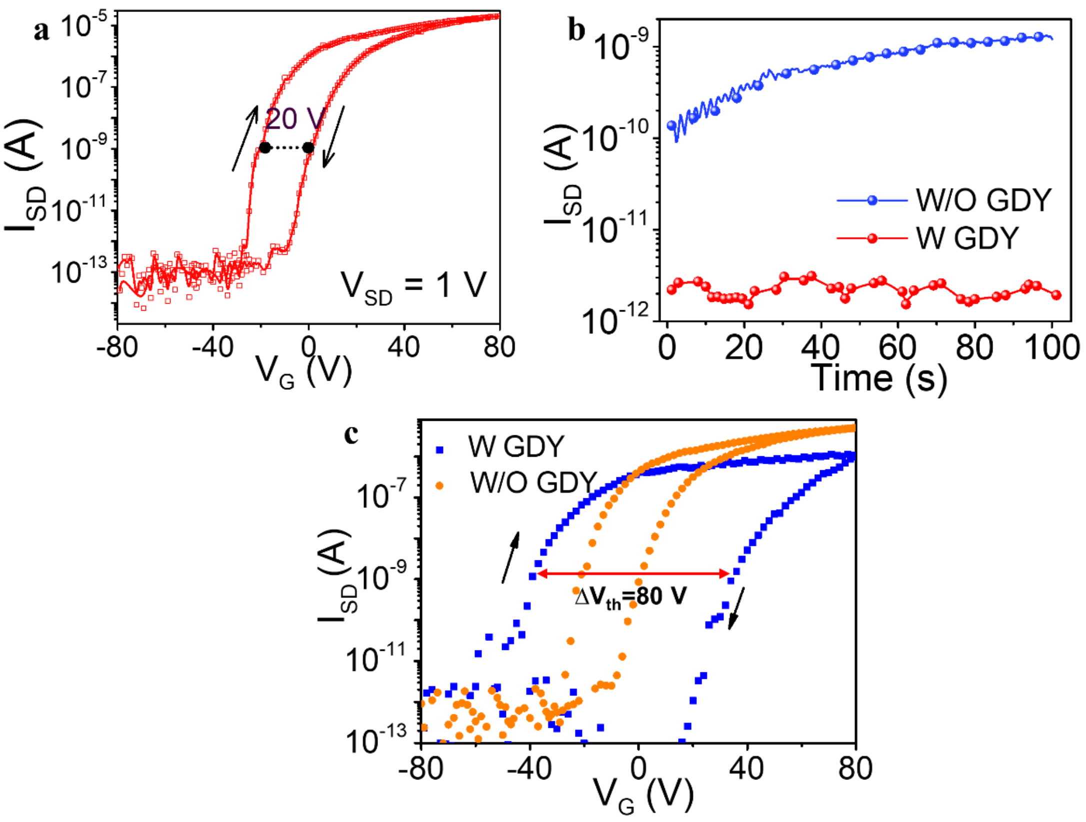

Revised Supporting Figure 5. Hysterisis effect of CVD-grown MoS_2 transistor on SiO_2, L=3 um, W=10 um. a) Transfer curve of the CVD-grown MoS_2 without GDY. b) Retention characteristics of a MoS_2 after reset operation which is partially transferred onto GDY. Before each retention test, the device was first applied a +80 V gate pulse of ~8 s, and then performed the reading operation with 1 V bias voltage and 0 V gate voltage. MoS_2 transistor without GDY can not reach a low off current or maintain data for as less as 100 s. On the contrary, a low current is read and last for a long time by MoS_2/GDY bilayer memory. c) Transfer curves for MoS_2 FET with and without GDY.

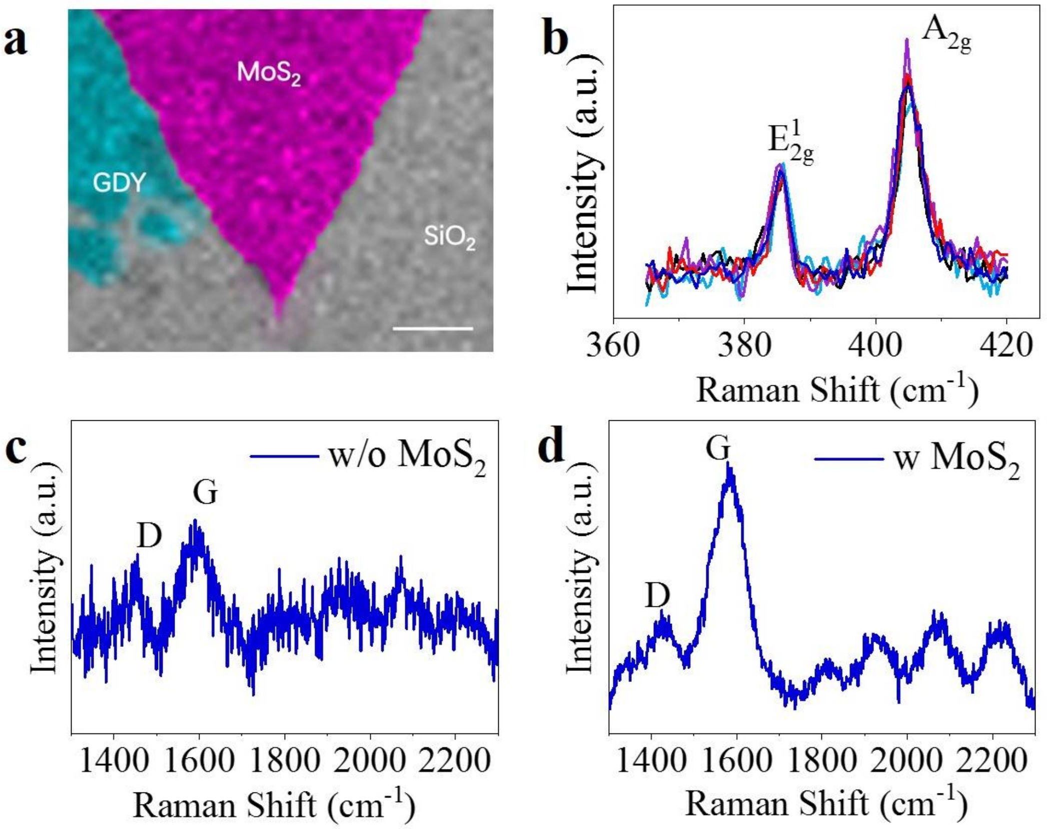

Supporting Figure 6. Raman mapping of MoS_2/GDY bilayer. a) SEM spectrum of MoS_2/GDY heterostructure. The area is coresponding to Raman mapping area in the main text (Fig.1). b) Five Raman spectrums from Raman mapping of Fig. 1c at range of MoS_2 on MoS_2/GDY bilayer. E^1_{2g} has no changed which indicates that MoS_2 is lying flat and parallel to GDY unstrainedly. c, d) Raman spectrums from Raman mapping of Figure 1d at range of GDY on MoS_2/GDY bilayer. The only difference between the raman measurement of (c) and (d) is that GDY film at (d) has a monolayer MoS_2 covered on the top. The measurement were made using a two accumulation of 5 s integration time and a 532 nm laser.

Both the Raman spectrum of (c) and (d) indicates the structure formation of GDY. The peaks of G, D and viration of -C≡C-C≡C- are observed which suggests the structure of GDY is unharmed. We also observed the D peak at 1432 cm^{-1} peak can be econvoluted into two peaks at 1367 and 1432 cm^{-1}. Peak at 1432 cm^{-1} refer to viration of C-C among triply coordinated atom and doubly coordinated neighbors.[5]

Comparing (c) and (d), however, we notice that bare GDY (Figure S4c) results in weak intensity, while GDY with MoS_2 covering above (Figure S4d) has intense and sharp bands. We attribute this to Raman scattering enhancement of MoS_2 towards GDY which is firstly reported. From G bands at 1582 cm^{-1} in this two spectras, we can obtain ratio of intensities as $I_{with}/I_{without}$=1022/249=4.1. The G band is chosen for calculation, because it is an important characteristic band of GDY and is distinguishable from interference with nearby lines. Monolayer MoS_2 enhanceing Raman signal of GDY is possibly attributed to its high oscilator strenth in exciton bands[6],[7] and charge transfer states formed with materials placed closely.[8] From the enhanced Raman spectrum, we caculate that the intensity ratio of D and G bands (D/G) is decreased to 0.39 which is smaller than original D/G ratio of original GDY. This verifies defects or amorphous structure are removed indicating that our mild oxygen plasma treatment is able to not only decrease the thickness, but also clean unwanted defects of GDY.

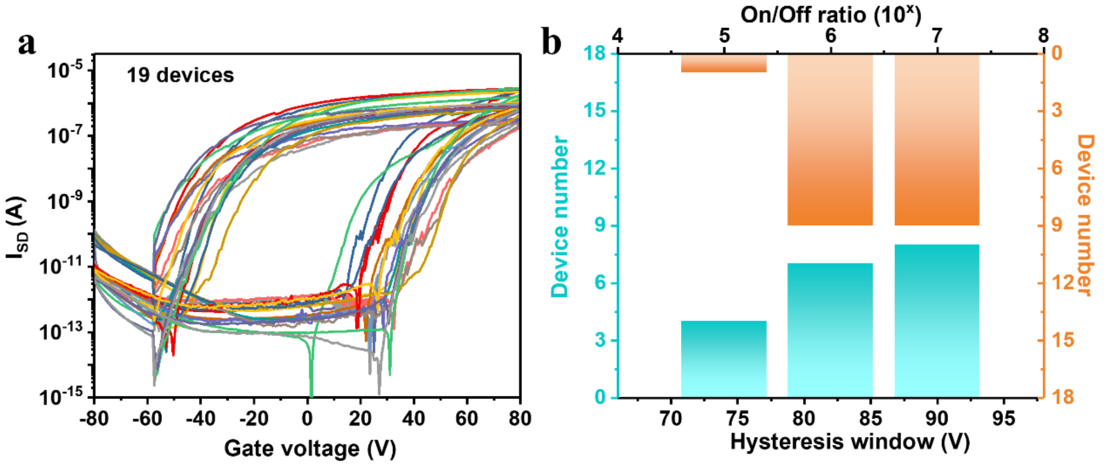

Supporting Figure 7. a). Transfer curves for 19 GDY/MoS_2 bilayer devices. b). Statistics of hysteresis window and On/Off ratio for 19 GDY/MoS_2 bilayer memory. All devices dimension are the same. L=3 um, W=10 um.

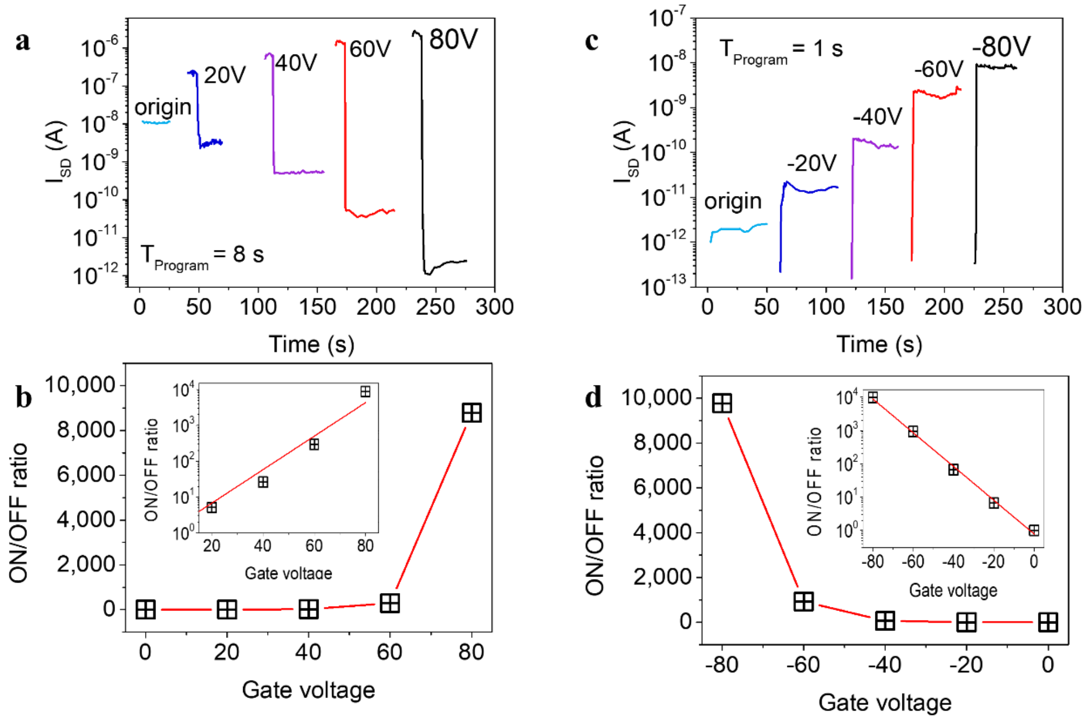

Supporting Figure 8. Influence of varying V_G on output behavior. a) The readout current as a function of V_{reset}. The reset time of V_{reset} is 5 s and then it is withdrawed for about 50 s. b) The ON/OFF ratio as a function of V_{reset}. The ON/OFF ratio is calculated by original current divided by resetting current. The inset was ON/OFF ratio (log scale) as a function of V_{reset}. The fitted line (red) indicates that the ON/OFF ratio is linearly dependent on the reset backgate. c) The readout current as a function of $V_{program}$ after reseting by a +80 V_{reset}. $V_{program}$ is operated for 1 s and then withdrawed for about 50 s. d) The ON/OFF ratio as a function $V_{program}$. The ON/OFF ratio is calculated by programming current divided by resetting current. The inset was ON/OFF ratio (log scale) as a function of $V_{program}$. The fitted line (red) indicates that the ON/OFF ratio is linearly dependent on $V_{program}$. Each plots of (a) and (c) is measured separated and put together with an interval of 15 s.

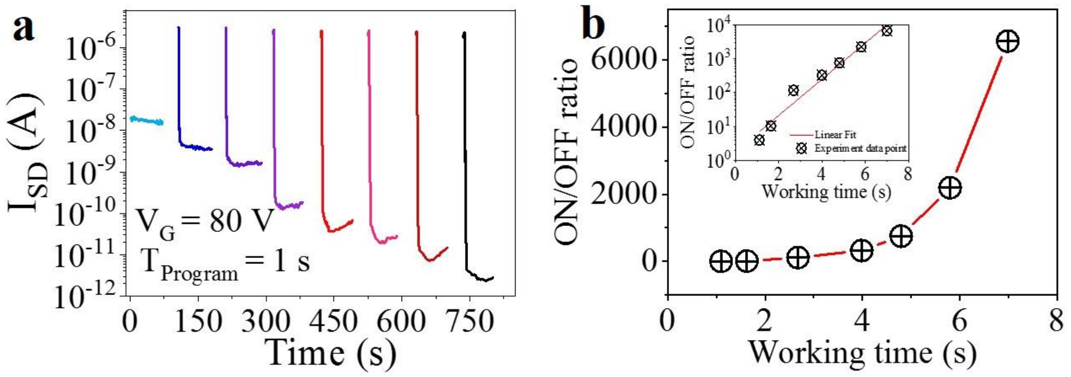

Supporting Figure 9. Influence of integrating time on output behavior. a) V_{reset} of 80 V is operated with 1 s and for 7 times. b) Linear dependence of the ON/OFF ratio on the gate programming time. It is implied that integration time can also be used to control the device state.

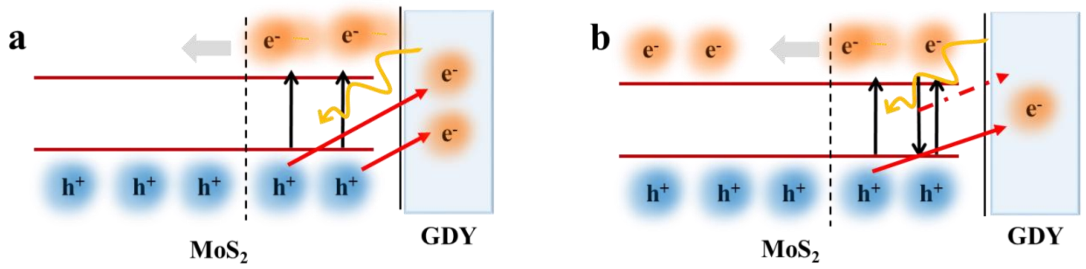

Supporting Figure 10. Schematic illustration of photoluminescence behavior of device. a) PL spectra evolution after applying a positive gate pulse. As electrons trapped in GDY after reset operation, blue shift of PL peak is observed because of the decreased A^- excitons and the increased A exciton as shown in main text Fig. 2. The decreased PL intensity is also observed. This suggests that photo generated holes and electrons are separated by the electric field between MoS_2 and GDY. In this process, photo generated holes transfer to GDY, while photogenerated electrons left in MoS_2 as carriers. b) PL recovering after applying a negative gate pulse. As the negative gate pulse release a part of trapped electrons from GDY to MoS_2, photogenerated holes and electrons are separated less which lead to red shift of PL peak and blue shift of PL intensity.

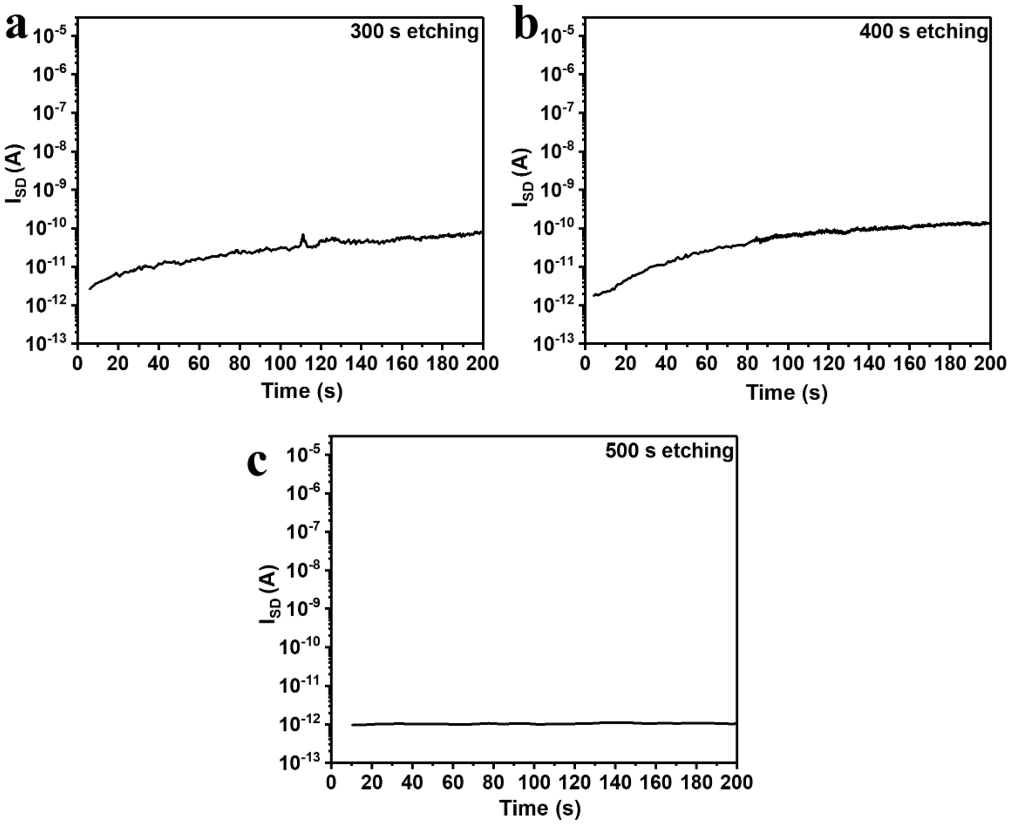

Supporting Figure 11. Retention measurements of devices with GDY of various time of oxygen plasma treatment. (a) 300 s, (b) 400 s and (c) 500 s etching time.

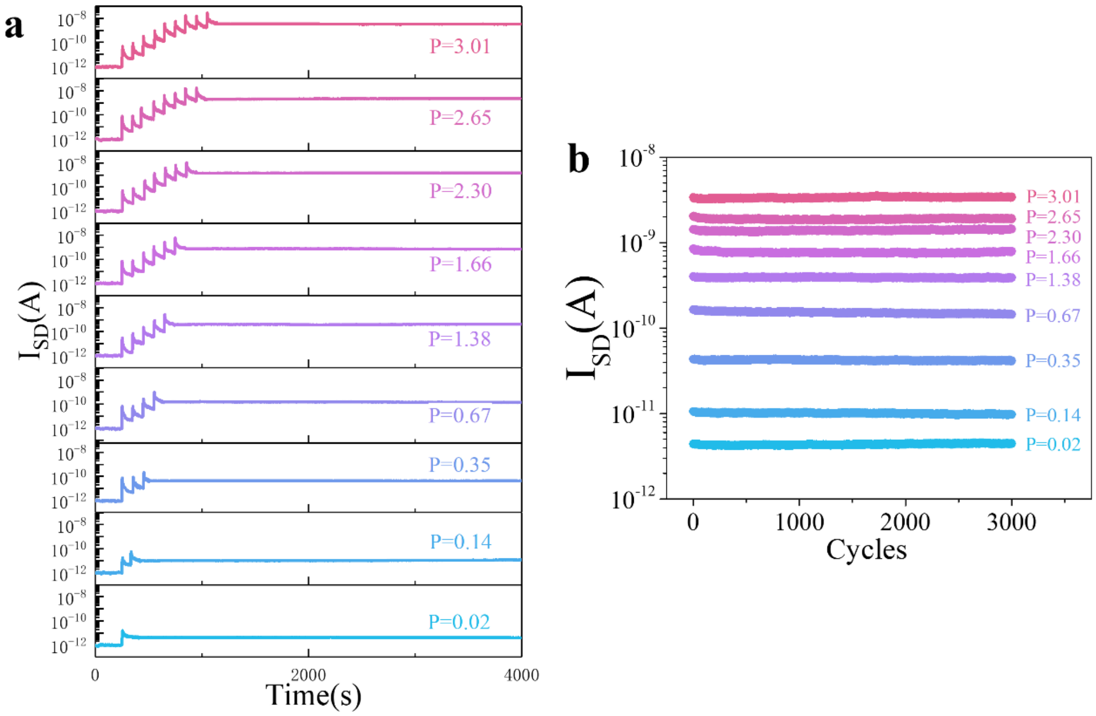

Supporting Figure 12. a) Retention time and b) Cyclic endurance performance of the GDY/MoS_2 memory in opto-electronic mode for $Light_{pro}$ pulses of intensity from 0.02 to 3.01 W/cm^2.

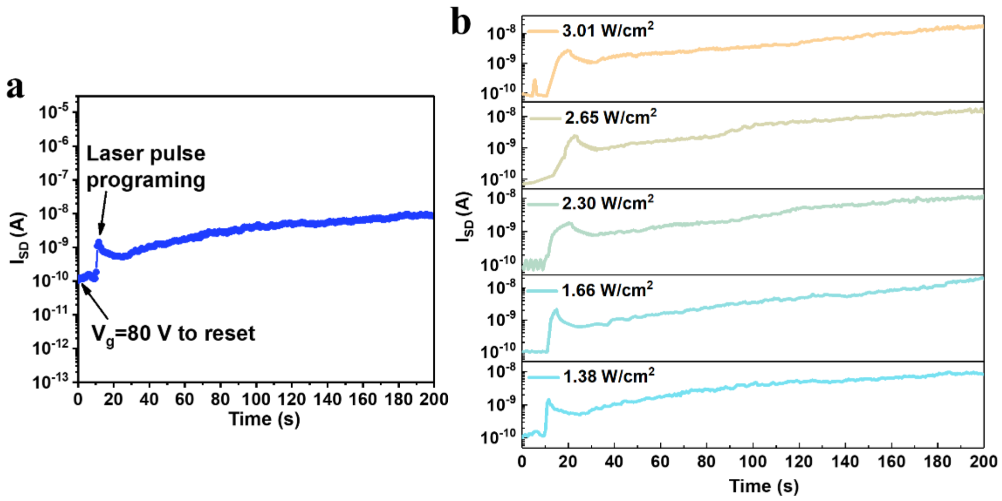

Revised Supporting Figure 13. Retention results after light programming with once laser pulse of (a) 1.38 W/cm2 for 1 s for the MoS_2 without GDY, which indicates the poor retention capability. (b) Comparison of retention for MoS_2 without GDY after laser pulse of various power for 1s. The MoS_2 FET has the gate length of 3 µm, and width of 10 µm.

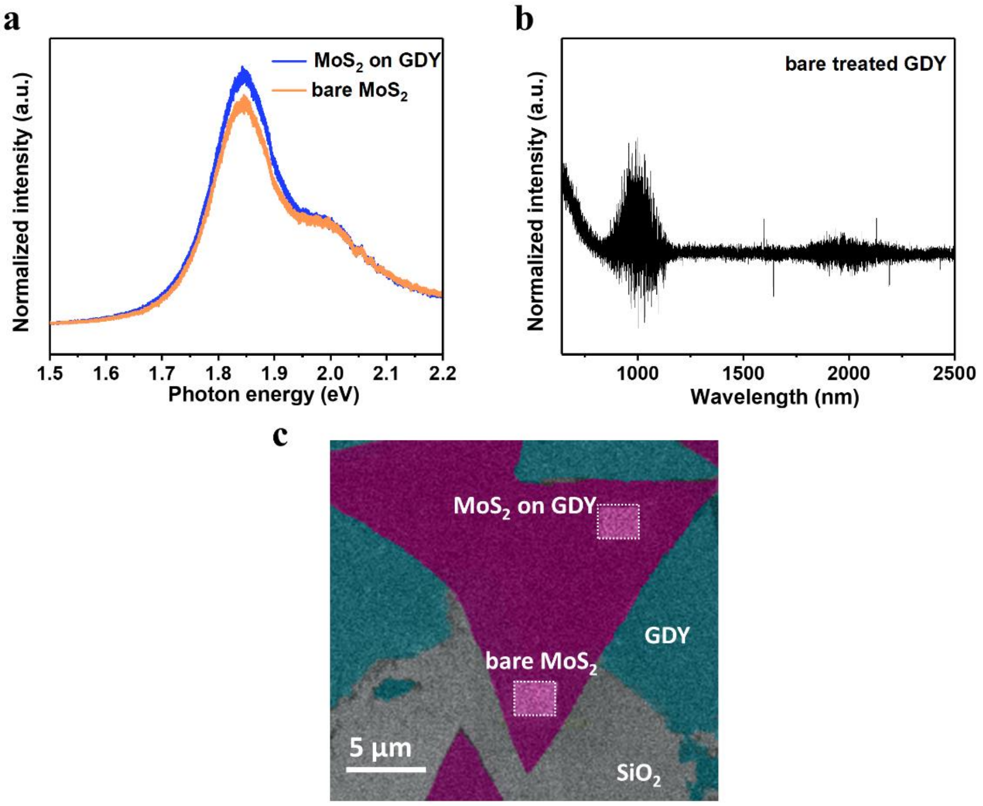

Supporting Figure 14. PL spectrum (a) from MoS_2 and MoS_2 on GDY and (b) from bare GDY after oxygen plasma treated. (c) The false color SEM image of GDY/MoS_2 bilayer heterostructure.

Supporting Table 1. Accuracy of multilevel readout current. Average current and standard deviation were calculated according to the data from Figure 3e. Ratio was calculated through dividing the standard deviation by the gap of measured average current and the current below.

State(n)	Average current of state n (In, A)	Standard deviation	Ratio
1	1.1325p	424.5333f	0.26051
2	5.6831p	760.9254f	0.46196
3	15.6095p	3.8246p	0.25697
4	54.1462p	6.07803p	0.3885
5	162.3065p	35.9421p	0.23827
6	520.7025p	49.4542p	0.35324
7	1.4264n	270.4867p	0.20852
8	5.7337n	627.6830p	0.51176
9	12.8226n	3.0001n	--

References

[1] G. Li, Y. Li, H. Liu, Y. Guo, Y. Li, D. Zhu, *Chem. Commun. (Camb)* **2010**, 46, 3256.

[2] J. Wang, S. Zhang, J. Zhou, R. Liu, R. Du, H. Xu, Z. Liu, J. Zhang, Z. Liu, *Phys. Chem. Chem. Phys.* **2014**, 16, 11303.

[3] H. Li, Q. Zhang, C. C. R. Yap, B. K. Tay, T. H. T. Edwin, A. Olivier, D. Baillargeat, *Adv. Funct. Mater.* **2012**, 22, 1385.

[4] A. Splendiani, L. Sun, Y. Zhang, T. Li, J. Kim, C. Y. Chim, G. Galli, F. Wang, *Nano Lett.* **2010**, 10, 1271.

[5] J. Zhou, Z. Xie, R. Liu, X. Gao, J. Li, Y. Xiong, L. Tong, J. Zhang, Z. Liu, *ACS Appl. Mater. Inter.* **2019**, 11, 2632.

[6] X. Ling, W. Fang, Y. H. Lee, P. T. Araujo, X. Zhang, J. F. Rodriguez-Nieva, Y. Lin, J. Zhang, J. Kong, M. S. Dresselhaus, *Nano Lett.* **2014**, 14, 3033.

[7] M. Bernardi, M. Palummo, J. C. Grossman, *Nano Lett.* **2013**, 13, 3664.

[8] C. Muehlethaler, C. R. Considine, V. Menon, W.-C. Lin, Y.-H. Lee, J. R. Lombardi, *ACS Photonics* **2016**, 3, 1164.
